# A study to explore the barriers to research utilization among staff nurses

**DOI:** 10.6026/9732063002001858

**Published:** 2024-12-31

**Authors:** Buvaneswari Ramakrishnan, Kowsalya D., Jamuna Rani P., Mahalakshmi B., Baskaran M., Siva Subramanian N., Ponmari K.

**Affiliations:** 1 Department of Psychiatric Nursing, KMCH College of Nursing, Coimbatore, Tamil Nadu - 641048, India; 2 Department of Pediatric Nursing, Nootan College of Nursing, Sankalchand Patel University, Visnagar, Gujarat - 384315, India; 3 Department of Psychiatric Nursing, PSG college of Nursing, Coimbatore, Tamil Nadu - 641004, India; 4 Department of Psychiatric Nursing, Nootan College of Nursing, Sankalchand Patel University, Visnagar, Gujarat - 384315, India; 5 Department of Medical surgical Nursing, SGRRIM & HS College of Nursing, Shri Gururam Rai University, Dehradun, Uttarakhand - 248001, Indi

**Keywords:** Research utilization, nursing, barriers, evidence-based practice

## Abstract

Research utilization (RU) in nursing is essential for improving patient outcomes by integrating evidence-based knowledge into clinical practice. Despite its significance, many nurses face barriers such as time constraints, lack of authority and inadequate organizational support, which hinder the effective application of research in practice. This study aims to identify the barriers preventing staff nurses from utilizing research findings in clinical settings. A cross-sectional descriptive study was conducted at a tertiary care hospital with 369 staff nurses. Data were collected using the Barriers to Research Utilization Scale and a Practice Questionnaire. Demographic variables and their association with research utilization were analyzed using chi-square tests. The study revealed that 55.83% of nurses had adequate research utilization practices, while 44.17% had inadequate practices. Organizational barriers, such as lack of authority (36.3%) and insufficient facilities (34.4%), were identified as key obstacles. Educational qualifications were significantly associated with better research utilization (p<0.001). Time constraints and limited access to research reports also emerged as major barriers. Organizational factors and educational qualifications play a significant role in research utilization among staff nurses. Enhancing organizational support and educational opportunities can help bridge the gap between research and practice, promoting evidence-based care.

## Background:

Research utilization (RU) in nursing refers to the application of study findings in clinical practice, aiming to improve patient outcomes by integrating new knowledge into everyday care. The importance of RU cannot be overstated, as it enhances the quality of care, promotes evidence-based practices and encourages innovative approaches that can lead to better patient outcomes [1]. Despite the availability of research in nursing, many practices still rely heavily on tradition and routine, rather than on scientific evidence. This gap between research and practice is a well-recognized challenge in healthcare settings globally [2]. Studies have repeatedly shown that this lack of control is a key obstacle in research utilization, making it difficult for nurses to translate research findings into practice [3]. Another critical barrier is the lack of time to engage with and apply research. Many nurses report that their workloads leave them with little time to read, comprehend, or implement new research findings in their day-to-day practice [4]. This time constraint is exacerbated by the complex and demanding nature of nursing roles, which prioritize immediate patient care over research engagement. As a result, nurses often struggle to stay updated on the latest evidence, which hinders the adoption of research into their clinical practice [5]. Their ability to integrate research findings into practice can significantly influence patient care quality [6].

The process of RU is often hindered by several barriers, including lack of time, resources and organizational support [7]. Some nurses may lack the necessary skills to critically appraise research or may not see its relevance to their daily clinical activities. These challenges underscore the need to identify and address the factors that impede RU, to foster a culture of evidence-based practice within nursing [8]. This study aims to explore the barriers that prevent staff nurses from utilizing research findings in clinical practice. Understanding these barriers is essential for developing strategies to enhance research utilization and ultimately improve patient care. By identifying the key obstacles faced by nurses, this study seeks to contribute to the growing body of knowledge on RU and help bridge the gap between research and practice in nursing.

## Methodology:

## Research design:

This quantitative, cross-sectional descriptive study aimed to explore barriers to research utilization among staff nurses.

## Setting:

The study was conducted in a large tertiary care hospital Pondicherry, covering various departments like Medicine, Surgery, Pediatrics and others.

## Participants:

A total of 369 staff nurses were selected through convenience sampling. Inclusion criteria [9, 10] included nurses aged 18 and above, working in clinical settings. Nurses in community health or unavailable during data collection were excluded.

## Instruments:

Data were collected using two tools:

[1] Barriers to research utilization scale: A 35-item Likert scale assessing barriers in four domains: nurse characteristics, organizational factors, research qualities and communication.

[2] Practice questionnaire: A nine-item scale measuring the application of research findings, with scores ranging from 0-27.

## Data analysis:

Descriptive statistics were used to summarize demographic data, while chi-square tests and t-tests explored associations between research utilization and demographic factors. Analysis was performed using SPSS version 20, with significance set at p<0.05.

## Results & Discussion:

**Figure 1:**
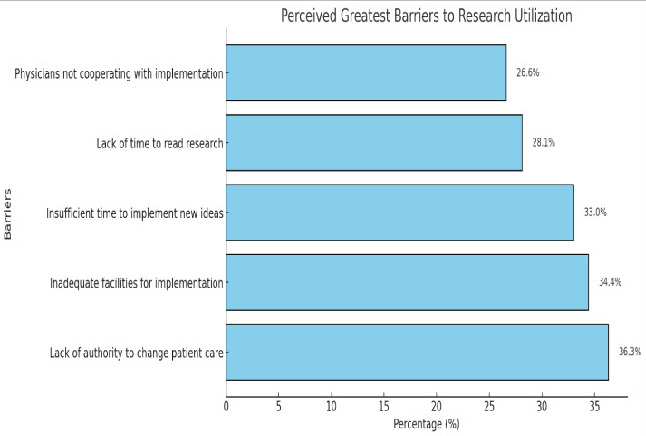
A pie chart showing the distribution of the sample as perceived major barriers to research utilization.

Table 1 shows majority of nurses were aged 20-30 years (79.94%) and female (71.54%). Most held a B.Sc. in nursing (76.42%) and were married (63.41%). Only 2.71% were actively involved in research. These demographic details provide context for analyzing barriers to research utilization. The analysis in Table 2 highlights that educational qualifications were significantly associated with research utilization (χ^2^ = 22.79, p < 0.001), indicating that nurses with higher qualifications, such as B.Sc. and M.Sc., were more likely to engage in evidence-based practices. However, other variables, including age (χ^2^ = 0.129, p = 0.719), gender (χ^2^ = 1.93, p = 0.165) and marital status (χ^2^ = 0.44, p = 0.509), showed no significant association with research utilization. These findings suggest that education plays a pivotal role in enhancing research engagement, while other demographic factors have limited influence. Figure 1 pie chart illustrates that organizational barriers were the most frequently cited obstacles, including lack of authority (36.3%) and inadequate facilities (34.4%). The present study found that 55.83% of staff nurses had adequate practice in updating their knowledge on research outcomes, while 44.17% showed inadequate practice. The primary barriers identified were organizational factors, such as a lack of authority to change patient care procedures (36.3%) and inadequate facilities for implementing research findings (34.4%). These findings suggest that organizational support plays a crucial role in enhancing research utilization among nurses, a conclusion consistent with previous studies [11]. Our results are in line with those of Wang *et al.* (2013), who found that nurses in a community hospital also cited organizational factors, such as lack of authority and time, as the greatest barriers to research utilization [12]. Similar results were reported by Jabonete *et al.* (2022), where nurses in Sweden perceived inadequate facilities and time constraints as major barriers to research utilization [13]. Both studies highlight the significant impact that workplace factors have on the ability of nurses to integrate research into their clinical practice. Our study found that educational qualification was significantly associated with research utilization, with nurses holding B.Sc. or M.Sc. degrees being more likely to engage in evidence-based practices. This finding is supported by Squires *et al.* (2011), who also found that nurses with higher educational qualifications exhibited greater research utilization [14]. Educational opportunities, therefore, play a critical role in empowering nurses to apply research findings in practice. In a study by Bahadori *et al.* (2016), time constraints were also cited as one of the most significant barriers to research utilization [15]. The similarity of findings across different settings indicates that lack of time remains a universal challenge for nurses in implementing research findings. Furthermore, Pitsillidou *et al.* (2021) highlighted that statistical complexities in research findings and inadequate support from senior colleagues were barriers to research implementation [16]. These communication challenges align with our findings, where inadequate access to research reports was mentioned as a barrier by a portion of the staff nurses. Chien *et al.* (2013) reported similar barriers, where time constraints, lack of authority and insufficient relevant research were the top perceived barriers among nurses [17]. Additionally, Tsai (2000) in the Republic of China found that organizational support, including access to resources and collaboration with researchers, was a key facilitator of research utilization [18]. These studies reinforce the need for healthcare organizations to improve access to research resources and foster a collaborative environment for nurses to thrive in evidence-based practice. Finally, a study by Almutairi *et al.* (2022) also emphasized the importance of organizational culture in research utilization. They found that providing nurses with learning opportunities, supportive environments and relevant resources significantly increased research utilization [1]. Our findings align with this, further underscoring the need for organizational changes that promote research-friendly environments to enhance the quality of care.

## Figures and Tables

**Table 1:** Demographic characteristics of staff nurses (n = 369)

**Variable**	**Frequency (n)**	**Percentage (%)**
**Age (years)**		
20-30	295	79.94
30-40	67	18.15
40-50	7	1.89
**Gender**		
Male	105	28.45
Female	264	71.54
**Educational Qualification**		
GNM	33	8.94
B.Sc. Nursing	282	76.42
M.Sc. Nursing	54	14.63
**Marital Status**		
Unmarried	135	36.58
Married	234	63.41
**Currently Involved in Research**		
Yes	10	2.71
No	359	97.29

**Table 2:** Association between demographic variables and level of practice on research utilization (n = 369)

**Demographic Variable**	**Chi-Square (χ^2^)**	**P-value**
Age (years)	0.129	0.719 (NS)
Gender	1.93	0.165 (NS)
Educational Qualification	22.79	<0.001 (S)
Marital Status	0.44	0.509 (NS)
Type of Family	0.13	0.715 (NS)
